# MIFAM-DTI: a drug-target interactions predicting model based on multi-source information fusion and attention mechanism

**DOI:** 10.3389/fgene.2024.1381997

**Published:** 2024-05-06

**Authors:** Jianwei Li, Lianwei Sun, Lingbo Liu, Ziyu Li

**Affiliations:** Institute of Computational Medicine, School of Artificial Intelligence, Hebei University of Technology, Tianjin, China

**Keywords:** drug-target interactions, multi-source information fusion, graph attention network, multi-head self-attention, fully connected layer

## Abstract

Accurate identification of potential drug-target pairs is a crucial step in drug development and drug repositioning, which is characterized by the ability of the drug to bind to and modulate the activity of the target molecule, resulting in the desired therapeutic effect. As machine learning and deep learning technologies advance, an increasing number of models are being engaged for the prediction of drug-target interactions. However, there is still a great challenge to improve the accuracy and efficiency of predicting. In this study, we proposed a deep learning method called Multi-source Information Fusion and Attention Mechanism for Drug-Target Interaction (MIFAM-DTI) to predict drug-target interactions. Firstly, the physicochemical property feature vector and the Molecular ACCess System molecular fingerprint feature vector of a drug were extracted based on its SMILES sequence. The dipeptide composition feature vector and the Evolutionary Scale Modeling -1b feature vector of a target were constructed based on its amino acid sequence information. Secondly, the PCA method was employed to reduce the dimensionality of the four feature vectors, and the adjacency matrices were constructed by calculating the cosine similarity. Thirdly, the two feature vectors of each drug were concatenated and the two adjacency matrices were subjected to a logical OR operation. And then they were fed into a model composed of graph attention network and multi-head self-attention to obtain the final drug feature vectors. With the same method, the final target feature vectors were obtained. Finally, these final feature vectors were concatenated, which served as the input to a fully connected layer, resulting in the prediction output. MIFAM-DTI not only integrated multi-source information to capture the drug and target features more comprehensively, but also utilized the graph attention network and multi-head self-attention to autonomously learn attention weights and more comprehensively capture information in sequence data. Experimental results demonstrated that MIFAM-DTI outperformed state-of-the-art methods in terms of AUC and AUPR. Case study results of coenzymes involved in cellular energy metabolism also demonstrated the effectiveness and practicality of MIFAM-DTI. The source code and experimental data for MIFAM-DTI are available at https://github.com/Search-AB/MIFAM-DTI.

## 1 Introduction

The development of new drugs is commonly associated with challenges such as high investment, high risks, long cycles, and low success rates. These challenges primarily stem from the complexity and uncertainty involved in drug discovery and development (Berdigaliyev and Aljofan, 2020), as well as stringent regulatory and requirements. In recent years, drug repositioning (Jourdan et al., 2020) has emerged as a highly promising approach in drug development. It involves repurposing approved drugs, originally intended for treating one disease, for the treatment of other distinct diseases. By repurposing existing drugs, the drug development cycle can be significantly shortened, and costs can be reduced. In addition, since the safety and side effects of these drugs have already been validated in previous clinical trials, the clinical trial risks of drug repurposing are greatly reduced. This not only expedites the development process but also provides a higher level of assurance in terms of safety and tolerability.

Accurate identification of potential drug-target interactions (DTIs) is critical for drug repurposing efforts (Anusuya et al., 2018). With the advancement of computer technology and the widespread accessibility of various relevant biological databases, computational methods (Talevi, 2018) have become indispensable tools for predicting and identifying DTIs. These methods utilize techniques such as machine learning, data mining, and network analysis. By integrating known information of drugs and targets, these models have been built to identify potential DTIs. Moreover, the widespread availability of public corresponding biological databases also provides critical support for drug repositioning. The drug databases like DrugBank (Knox et al., 2024) and PubChem (Kim, 2016), as well as target databases like UniProt (Zaru and Orchard, 2023) and NCBI (O’Leary et al., 2016), offer rich information about drugs and targets, including chemical structures, biological activities, interaction networks, etc. Researchers can leverage the data from these databases for comprehensive analysis and discover new drug-target interactions. To date, numerous prediction methods and models have been proposed to uncover potential DTIs, which can be divided into three categories based on their data sources and execution algorithms.

The first category of DTI prediction methods is the structure-based methods (Fauman et al., 2011), which utilize the molecular structure of drugs and the spatial structure of target proteins as inputs to identify the drug-target interactions. These methods rely on techniques such as molecular docking and structure alignment (Ferreira et al., 2015) to predict how drugs bind to targets. However, the application of them in large-scale DTI prediction is limited by the fact that the three-dimensional structures of the majority of target proteins with known sequences are still unknown. The second category is ligand-based methods (Pozzan, 2006), which use known information about drug-target binding ligands to predict the binding of new drugs to similar targets. These methods compare the binding ligands of drugs to known targets and make predictions based on similarity. However, they heavily depend on the availability of known binding ligand information and may not be suitable for new drugs or targets without known binding ligands. The third category encompasses methods based on machine learning or deep learning (Askr et al., 2023). These methods employ algorithms to learn patterns and rules from large amounts of drug and target data, enabling the prediction of drug-target interactions. They can utilize existing drug-target interaction networks and bioinformatics features for feature extraction and pattern recognition. Machine learning and deep learning methods offer flexibility and predictive capabilities, allowing for DTI prediction even in the absence of structural or ligand information. These methods have demonstrated good accuracy and robustness on various datasets. As bioinformatics features become more readily available and datasets expand, machine learning methods are gradually being replaced by deep learning methods. Deep learning methods can automatically learn features and capture more accurate patterns and rules from large datasets, making them more effective for DTI prediction (Schauperl and Denny, 2022).

In recent years, an increasing number of machine learning and deep learning models for DTI prediction have been developed and have achieved excellent prediction performance. Yuan et al. (2016) developed a novel machine learning method called DrugE-Rank, which improved prediction performance by calculating the chemical similarity between input compounds and known active compounds. Lee and Nam (2018) introduced a new DTI prediction method called RWR, which utilized global network topology information and a random walk with restart (RWR) algorithm to simulate drug-target interaction and predict untested DTIs. Wan et al. (2019) presented a deep learning model named DeepCPI, which employed multi-layer convolutional neural networks (CNNs) and recurrent neural networks (RNNs) to extract features from drugs and targets. After feature extraction, the model merged the drug and target features and made predictions through fully connected layers. Lee et al. (2019) proposed a deep learning model called DeepConv-DTI based on convolutional neural networks, which extracted features from the structural representation of drug molecules and the sequence information of target proteins using multiple layers of CNNs. After feature extraction, the model combined the drug and target features and predicted DTI results through fully connected layers. Chen et al. (2020) proposed a novel model called TransformerCPI, which utilized attention mechanisms capable of learning feature weights to assess the importance of different atoms. Additionally, this model employed parallel computing techniques to reduce the computational complexity. A deep learning model called MHSADTI was developed by Cheng et al. (2022) which utilized graph attention networks (GATs) and multi-head self-attention (MHSA) to better extract features from drugs and proteins. The drug and protein feature vectors were concatenated and fed into fully connected layers for final result prediction. A deep learning model called AMMVF-DTI was developed by Wang et al. (2023), which combined multimodal and multi-view information and incorporated attention mechanisms for feature fusion to enhance prediction accuracy and reliability.

In this study, our objective was to develop a novel end-to-end deep learning model named MIFAM-DTI, which integrated multi-source information fusion and attention mechanisms to enhance the accuracy of DTI prediction. To effectively extract and retain the feature information of drugs and targets, the MIFAM-DTI model incorporated graph attention networks and multi-head self-attention, both of which were enriched with attention mechanisms. First, the physicochemical property (PCP) feature vector (Raevsky, 2004) and the Molecular ACCess System (MACCS) molecular fingerprint feature vector of drugs were computed based on their SMILES sequences. For target proteins, the feature vector of dipeptide composition (DC) and the Evolutionary Scale Modeling -1b (ESM-1b) feature vector (Rives et al., 2021) based on their amino acid sequences were calculated. As the dimensions of the four feature vectors may be different and contain noisy data, Principal Component Analysis (PCA) was adopted to reduce each feature vector to 128 dimensions for calculating the adjacency matrices of the four feature vectors based on cosine similarity. Next, the two drug feature vectors were concatenated, and a logical OR operation was performed on the two adjacency matrices. The concatenated drug feature vector and drug adjacency matrix were then fed into the deep learning model, which comprised graph attention networks and multi-head self-attention. This process resulted in the final drug representation vector. The same operations were repeated for target data to obtain the final representation vector of targets. Finally, the final representation vectors of drugs and targets were concatenated and input into a multilayer perceptron (MLP) composed of fully connected layers and users could obtain the predicted scores of drug-target interactions. To evaluate the performance of MIFAM-DTI, we conducted experiments on two datasets, namely, *C. elegans* and Human. The results demonstrated that our model outperformed existing state-of-the-art methods in terms of AUC and AUPR, which are commonly used evaluation metrics for DTI prediction. Furthermore, to further validate the effectiveness of our model, we conducted a case study focusing on coenzyme-like substances involved in cellular energy metabolism.

## 2 Materials and methods

### 2.1 Datasets

In supervised learning tasks pertaining to DTI prediction, the dataset often comprises positive samples representing confirmed drug-target interactions and negative samples representing the absence of such interactions. However, an imbalanced distribution of negative samples in the experimental dataset, either with an excess or a scarcity, can lead to inaccurate results, reduced recall rate, model overfitting, and impaired generalization. Therefore, the careful selection of appropriate negative samples to construct the dataset is crucial for ensuring reliable experimental results.

In this study, we employed two benchmark datasets, namely, *C. elegans* and Human, which were originally generated by Liu et al. (2015). To ensure the integrity of our evaluation, the duplicate entries from these datasets were eliminated. The positive samples in both datasets were derived from the DrugBank and Matador databases, which are reputable sources of information on drug-target interactions. As for the negative samples, they adopted a meticulous approach involving multiple iterations of the classifier, gradually selecting a highly reliable set of negative samples. The *C. elegans* dataset, documented in Supplementary Table S1, encompasses 3,893 positive interactions involving 1,434 compounds and 2,504 proteins. On the other hand, the Human dataset, detailed in Supplementary Table S2, comprises 3,364 positive interactions between 1,052 compounds and 852 proteins. It is noteworthy that the ratio of positive to negative samples is maintained at 1:1 in both datasets, ensuring a balanced representation of different classes. For further insights, Table 1 provides comprehensive information regarding these two datasets, including their key properties.

**TABLE 1:** Summary of the two datasets used in this study, *C. elegans* and human.

Datasets	*C. elegans*	Human
Number of drugs	1,434	1,052
Number of proteins	2,504	852
Number of total samples	7,786	6,728
Number of positive interactions	3,893	3,364

### 2.2 The PCP feature vector of drugs

**FIGURE 1:**
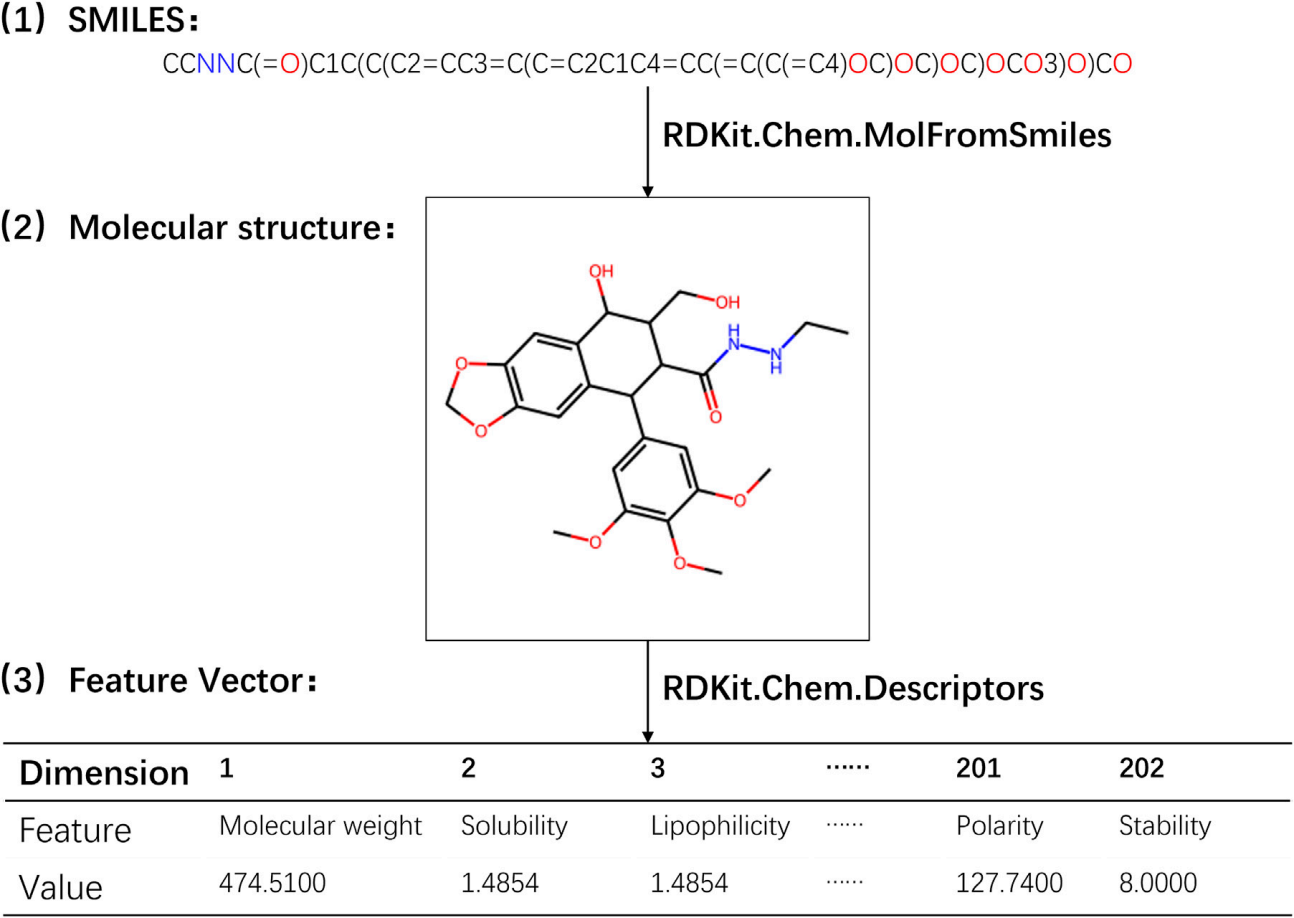
Flowchart for extracting the physicochemical property feature vector of drugs.

In the experiment, the calculation of physicochemical property feature vectors for drugs was performed using RDKit, an open-source cheminformatics toolkit widely utilized in computational chemistry research, molecular modeling, and drug discovery. RDKit offers a comprehensive range of tools and algorithms for handling and analyzing chemical molecules, encompassing molecular descriptor calculation, molecular transformation, molecular fingerprinting, molecular alignment, chemical reaction simulation, chemical data visualization, and more. To derive the physicochemical property features of drug molecules, a set of functions provided by the Descriptors module in RDKit was employed. These functions enable the calculation of various physicochemical properties, including molecular weight, solubility, polarity, and other relevant information (Priya et al., 2022). By applying these functions to the drug molecular object, we obtained 202 different eigenvalues of physical and chemical properties. Subsequently, these eigenvalues were connected in series to form a 202-dimensional physicochemical property feature vector for the drug. A detailed illustration of the process involved in obtaining the physicochemical property feature vector for drugs is presented in Figure 1 (refer to Supplementary Table S3).

Through the utilization of RDKit’s Descriptors module, we were able to efficiently compute the physicochemical property feature vectors, which encapsulate important characteristics of the drug molecules. These feature vectors served as valuable inputs for subsequent analysis and modeling in our study.

### 2.3 The MACCS molecular fingerprint feature vector of drugs

The MACCS molecular fingerprint is a commonly employed drug molecular descriptor for representing chemical structural features of drugs (He, 2022). It utilizes a binary encoding scheme, where each bit within the fingerprint corresponds to a specific structural fragment or substructure. The MACCS molecular fingerprint consists of 166 predefined structural features, along with an additional dimension, resulting in a total of 167 dimensions. These 166 predefined structural features encompass common chemical fragments, bonds, and rings. Each feature is encoded using a binary bit, with the presence of the corresponding structural element in the molecule denoted by a 1, while its absence is represented by a 0. The additional dimension is utilized to handle unknown or incorrect structures. In cases where certain structural elements within the drug molecule cannot be accurately encoded using the known structural features, they are assigned a value of 1 in the extra dimension, indicating the presence of unknown or erroneous structures.

**FIGURE 2:**
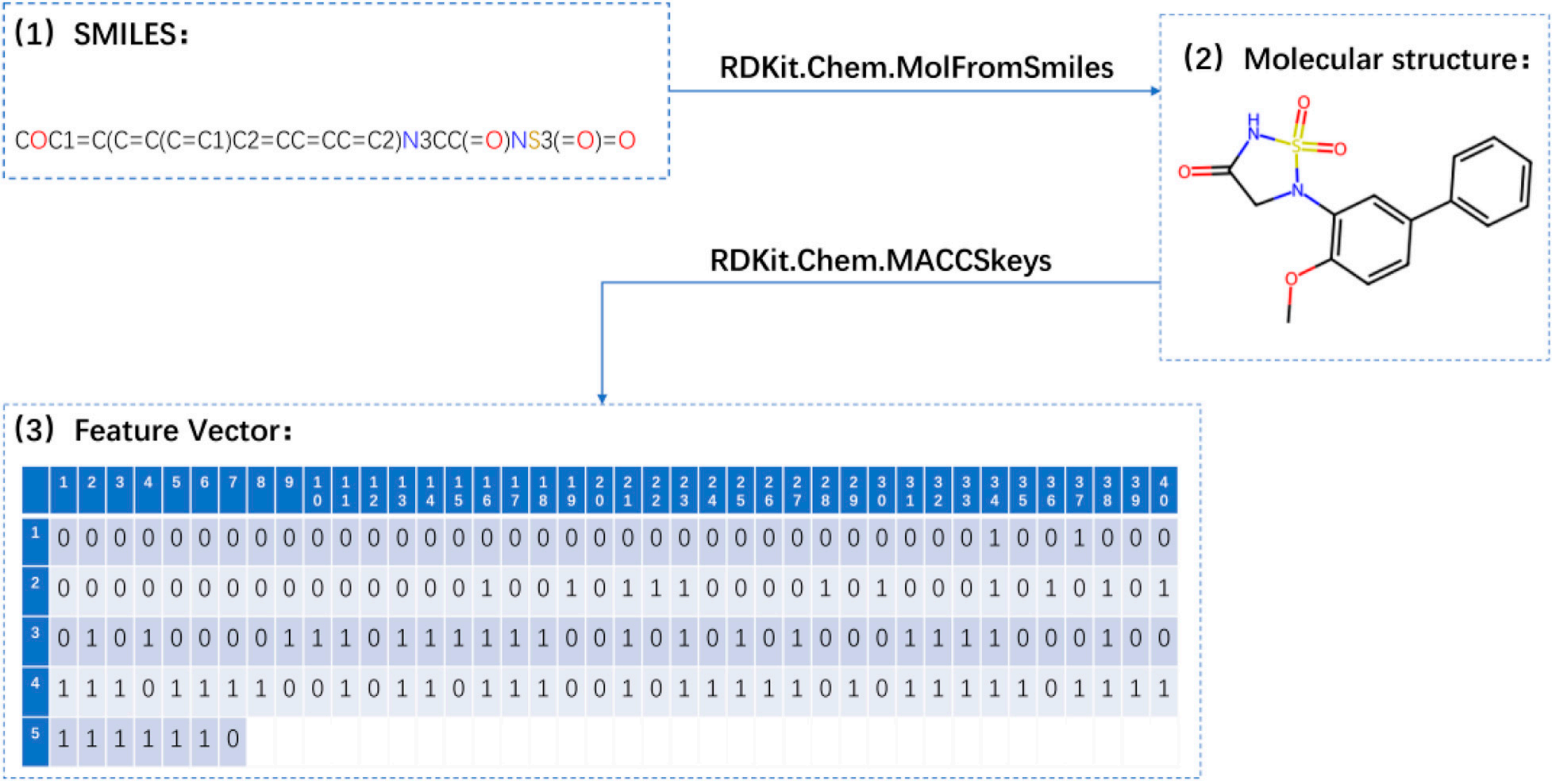
Flowchart for extracting the MACCS molecular fingerprint feature vector of drugs.

In this study, the MolFromSmiles function from the RDKit toolkit was employed to convert the drug’s SMILES sequence into a molecular object. This conversion process facilitated the structured representation and subsequent analysis of the drug. Subsequently, the MACCSkeys function, also available within the RDKit toolkit, was utilized to calculate the molecular fingerprint feature vector for the drug (refer to Supplementary Table S4). This feature vector encompasses both structural and property information relevant to the drug. By computing the similarity between fingerprints, the interaction relationships between drugs were inferred. A comprehensive depiction of the process involved in extracting the MACCS molecular fingerprint feature vector for the drug is presented in Figure 2.

By effectively utilizing the capabilities offered by the RDKit toolkit, the computation of MACCS molecular fingerprint feature vectors for the drug molecules was efficiently achieved, capturing crucial structural information. These feature vectors, incorporating both structural and property details, played a pivotal role in the analysis and modeling endeavors undertaken within this study.

### 2.4 The DC feature vector of targets

There are a total of 400 possible combinations of the 20 natural amino acids. Therefore, the amino acid sequence P of each protein can be represented by a 400-dimensional feature vector $V_{DC}\left(P\right)$ . In this experiment, we considered not only adjacent amino acid pairs in the sequence, but also include pairs of amino acids with one amino acid in between. Therefore, the feature vector $V_{DC}\left(P\right)$ had a dimensionality of 800 (see Supplementary Table S5). The calculation formula is shown in Eq. 1:

$$V_{DC}\left(P\right)=\left(f_{gap0-1},f_{gap0-2},\cdots ,f_{gap0-400},f_{gap1-1},f_{gap1-2},\cdots f_{gap1-400}\right)$$ (1)

where gap0 represents adjacent amino acid pairs, gap1 represents amino acid pairs with one amino acid in between, and $f_{gapi-j}\;\left(i=0,1\;j=1,2,\cdots ,400\right)$ represents the frequency of occurrence of each amino acid pair j in the protein sequence P .

### 2.5 The ESM-1b feature vector of targets

In this experiment, the ESM-1b version of the ESM model was selected. ESM-1b, developed by DeepMind in 2022, represents an advanced machine learning model specifically designed for protein structure prediction. With its extensive architecture comprising 650 million parameters and 33 layers, ESM-1b stands out as one of the largest protein language models currently available.

The ESM-1b model leverages neural networks and employs self-supervised learning techniques, capitalizing on vast protein sequence and structure databases for training purposes. Its fundamental principle lies in the utilization of the Transformer network to represent protein sequences and structures. The Transformer network architecture incorporates a self-attention mechanism, facilitating interactions between each position within the sequence and other positions. This mechanism empowers the model to acquire meaningful representations of protein sequences, effectively capturing crucial features and patterns (Wang et al., 2023). By employing self-attention, the ESM-1b model can effectively capture long-range dependencies and contextual information, thereby enhancing its capacity to make accurate predictions regarding protein structure. Furthermore, the ESM-1b model undergoes a pre-training phase using self-supervised learning on a substantial amount of unlabeled protein sequences (Zhou et al., 2023). During this phase, the model learns valuable features by predicting the relationships between different positions within the sequence. Additionally, the model leverages the evolutionary information inherent in protein sequences. By performing multiple sequence alignments of protein sequences from related species, the model captures co-evolutionary relationships among these sequences. The incorporation of evolutionary information enhances the model’s ability to predict protein structure, as related protein sequences often exhibit structural similarities.

First, we downloaded the pre-trained ESM-1b model to our local environment and set up the necessary experimental environment (For details, see https://github.com/facebookresearch/esm). Then, the amino acid sequences of target proteins from the *C. elegans* dataset and the Human dataset were fed into the model for training. Finally, we obtained the ESM-1b feature vector for each protein (see Supplementary Tables S6, S7).

### 2.6 Fusion of feature vectors

In order to fully retain the original feature information of the drugs and the targets, PCA dimensionality reduction to 128 dimensions was performed on the PCP feature vector, the MACCS molecular fingerprint feature vector, the DC feature vector and the ESM-1b feature vector obtained in the above steps. Then, the two feature vectors of the drugs and the two feature vectors of the targets were concatenated and fused respectively to obtain the drug fusion feature vector and the target fusion feature vector.

For the concatenation fusion operation, it was assumed that the PCP feature vector of drugs and the MACCS molecular fingerprint feature vector of drugs after dimensionality reduction were $X\in R^{n\times m}$ and $Y\in R^{n\times k}$ , where n was the number of drug samples, m was the dimension of the PCP feature vector, and k was the dimension of the MACCS molecular fingerprint feature vector. By combining the two eigenvector matrices X and Y on the column dimension, we obtained the drug fusion feature vector $Drug\in R^{n\times \left(m+k\right)}$ . The concatenation operation is shown in Eq. 2.

$$Drug=\left[X\left\|Y\right.\right]=\left[\begin{array}{ccc} X_{1,1} & \cdots & X_{1,m}\\ \vdots & \ldots & \vdots \\ X_{n,1} & \ldots & X_{n,m} \end{array}\;\begin{array}{ccc} Y_{1,1} & \ldots & Y_{1,k}\\ \vdots & \ldots & \vdots \\ Y_{n,1} & \ldots & Y_{n,k} \end{array}\right]$$ (2)

Similarly, the target fusion feature vector $Target\in R^{n^{\prime }\times \left(m^{\prime }+k^{\prime }\right)}$ was obtained by concatenating the DC feature vector with the ESM-1b feature vector. In the statement, $n^{\prime }$ represented the number of samples for the target, $m^{\prime }$ represented the dimensionality of the DC feature vector, and $k^{\prime }$ represented the dimensionality of the ESM-1b feature vector.

### 2.7 Calculation of similarity matrix

Cosine similarity serves as a widely utilized similarity measure for comparing the degree of similarity between two vectors. It quantifies the similarity by computing the cosine value of the angle formed between the vectors. One notable advantage of cosine similarity is its resilience to the dimensionality of the vectors, rendering it suitable for assessing similarity even when dealing with highly sparse vectors. Moreover, the simplicity and efficiency of cosine similarity calculations make it well-suited for large datasets (Zhou et al., 2021).

Prior to computing the cosine similarity, it becomes necessary to normalize the vectors by converting them into unit vectors. This normalization step is essential as cosine similarity primarily focuses on the directional aspect of vectors rather than their magnitude (Xie et al., 2021). The calculation of cosine similarity is represented by Eq. 3:

$$S\left(A,B\right)=\frac{A∙B}{\left\|A\right\|\times \left\|B\right\|}=\frac{\sum_{i=1}^{n}\left(A_{i}\times B_{i}\right)}{\sqrt{\sum_{i=1}^{n}{A_{i}}^{2}}\times \sqrt{\sum_{i=1}^{n}{B_{i}}^{2}}}$$ (3)

where $S\left(A,B\right)$ represents the cosine similarity between vector A and vector B . A∙B denotes the dot product of vectors A and B , $\left\|A\right\|$ represents the magnitude of vector A , and $A_{i}$ denotes the i -th component of vector A . The cosine similarity ranges from −1 to 1, where 1 indicates complete similarity, −1 indicates complete dissimilarity, and 0 indicates no correlation. A higher value indicates a higher similarity.

### 2.8 Graph attention network

The graph attention network (GAT) stands as a deep learning model specifically designed for analyzing graph data, with a particular focus on node-level tasks like node classification and node attribute prediction. Distinguishing itself from traditional graph convolutional neural networks, the GAT model assigns distinct attention weights to each neighbor of a node (Keicher et al., 2023). Consequently, each node possesses the ability to dynamically adjust its attention towards neighboring nodes based on its own features and the features exhibited by its neighbors. The model’s foundation lies in its attention mechanism, enabling it to autonomously learn the importance weights associated with the relationships between each node and its neighboring nodes (Zhao et al., 2022).

Similar to other attention mechanisms, the computation within the GAT model involves two primary steps: the calculation of attention coefficients and weighted summation. Initially, for a given node i , we computed the similarity coefficients with its neighboring nodes $\left(j\in N_{i}\right)$ and itself. The calculation formula is shown as Eq. 4:

$$e_{ij}=a\left(\left[Wh_{i}\left\|Wh_{j}\right.\right]\right),j\in N_{i}$$ (4)

where $e_{ij}$ represents the importance of node j with respect to node i , $N_{i}$ is the set of neighboring nodes of node i , [ ‖ ] denotes the concatenation operation applied to the features of nodes i and j . Finally, $a\left(∙\right)$ maps the concatenated high-dimensional features to a scalar value. Next, we need to normalize the values using the softmax function to obtain the attention coefficient $a_{ij}$ . The calculation process is shown in Eq. 5:

$$\alpha_{ij}=softmax\left(e_{ij}\right)=\frac{\exp\left(LeakyReLU\left(e_{ij}\right)\right)}{\sum_{k\in N_{i}}\exp\left(LeakyReLU\left(e_{ik}\right)\right)}$$ (5)

**FIGURE 3:**
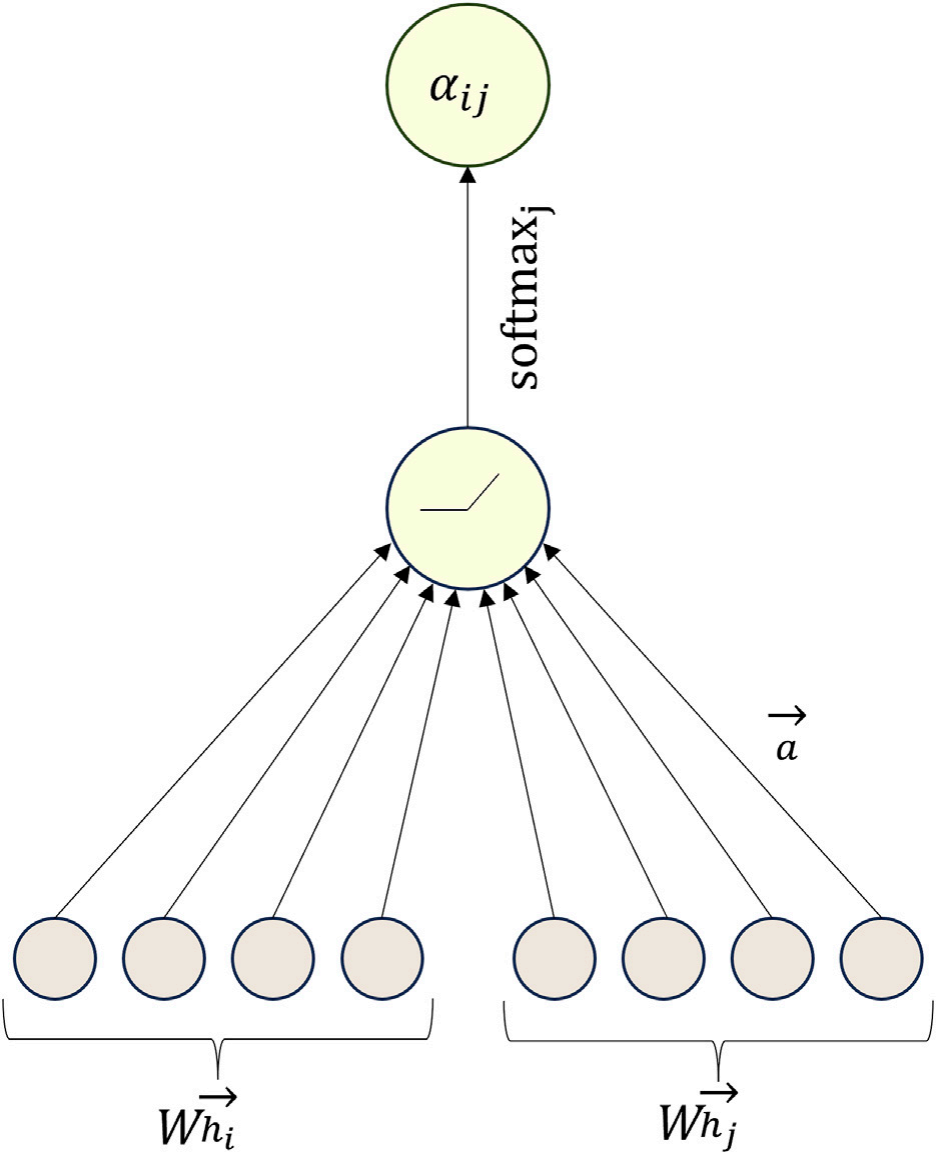
Example diagram of the calculation of attention coefficient $a_{ij}$ .

The steps for calculating the attention coefficient $a_{ij}$ can be understood by referring to Figure 3.

After calculating the attention coefficients, we need to perform a weighted summation of the features. The summation formula is shown as Eq. 6:

$$h_{i}^{\prime }=\sigma \left(\sum_{j\in N_{i}}\alpha_{ij}Wh_{j}\right)$$ (6)

where $\sigma \left(∙\right)$ represents the ReLU activation function, W is the dimension transformation matrix, $h_{j}$ is the feature vector of the j -th neighboring node, and $h_{i}^{\prime }$ is the new feature of node i calculated by the GAT algorithm. During the computation, the new feature of node i is represented as a weighted sum of the features of all its neighboring nodes.

To make the learning process of the attention mechanism more stable and effective, we introduce the concept of multi-head attention. This involves calculating new features using multiple attention mechanisms and concatenating the resulting features to obtain the final feature vector. As shown in Eq. 7:

$$h_{i}^{\prime \prime }=\begin{array}{c} K\\ \|\\ k=1 \end{array}h_{i}^{\prime^{k}}=\begin{array}{c} K\\ \|\\ k=1 \end{array}\sigma \left(\sum_{j\in N_{i}}\alpha_{ij}^{k}W^{k}h_{j}\right)$$ (7)

**FIGURE 4:**
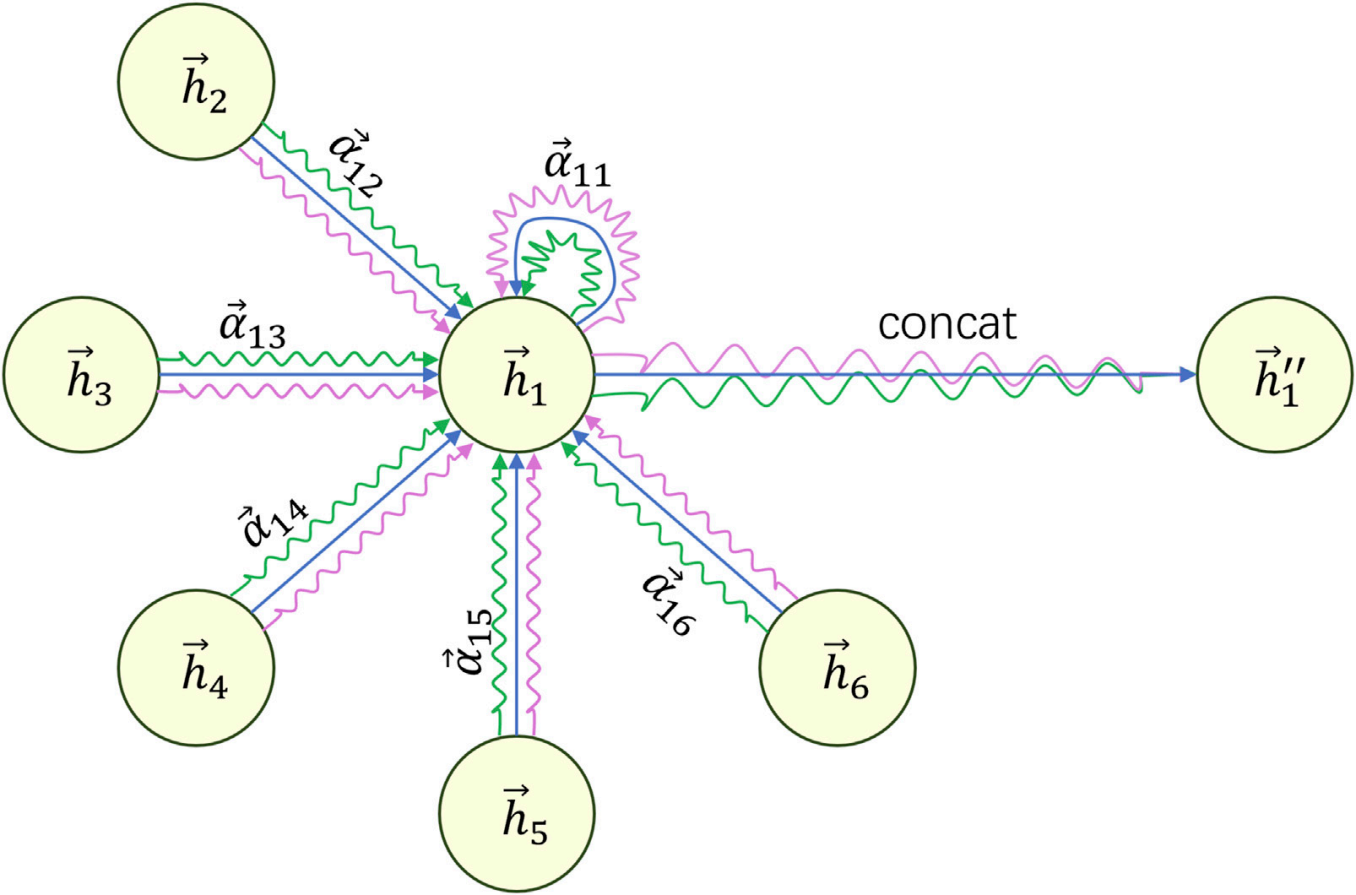
Example diagram of calculating the final features based on multi-head attention.

Where $h_{i}^{\prime \prime }$ represents the final feature vector after concatenation, K represents the number of attention heads, ‖ denotes the concatenation operation applied to the new features $h_{i}^{\prime }$ of K nodes, resulting in a final feature vector of dimension $K\times \dim\;\left(h_{i}^{\prime }\right)$ . The equation can be understood by referring to Figure 4.

### 2.9 Multi-head self-attention

Multi-head self-attention (MHSA) represents a prominent form of multi-head attention mechanism extensively employed for the analysis of sequential data, particularly in natural language processing (NLP) applications encompassing machine translation, text classification, and semantic understanding (Wang et al., 2021). It serves as an extension and enhancement of the conventional self-attention mechanism. MHSA introduces multiple heads, each associated with distinct attention weight matrices, to augment the model’s expressive capabilities (Deng et al., 2022).

In the traditional self-attention mechanism, for each element in the input sequence, it computes a query vector Q , a key vector K , and a value vector V . These vectors are obtained by linear transformations of the input elements using weight matrices learned during training. It is assumed that the input sequence is $X=\left[x_{1},x_{2},\cdots ,x_{n}\right]$ , and the weight matrices are $W_{Q}$ , $W_{K}$ , and $W_{V}$ . For each element $x_{i}$ , the calculation formulas for it are shown as Eq. 8:

$$Q_{i}=W_{Q}∙x_{i},\;K_{i}=W_{K}∙x_{i},\;V_{i}=W_{V}∙x_{i}$$ (8)

where $Q_{i}$ , $K_{i}$ , and $V_{i}$ represent the query vector, key vector, and value vector of the i -th element, respectively. Afterwards, it can compute the attention score between element $x_{i}$ and $x_{j}$ . This attention score is computed as the dot product of the query vector and the transpose of the key vector, divided by a scaling factor (usually the square root of the dimension of the key vector, i.e., $\sqrt{d_{K}}$ ). The calculation formula is given by Eq. 9:

$$score\left(Q_{i},K_{j}\right)=\frac{Q_{i}∙{K_{j}}^{T}}{\sqrt{d_{K}}}$$ (9)

Next, it normalizes the attention scores using the softmax function to obtain attention weights $w_{ij}$ , which are transformed into values between 0 and 1. The computation process for $w_{ij}$ is shown as Eq. 10:

$$w_{ij}=softmax\left(score\left(Q_{i},K_{j}\right)\right)=softmax\left(\frac{Q_{i}∙{K_{j}}^{T}}{\sqrt{d_{K}}}\right)$$ (10)

Finally, it multiplies each element’s value vector with its corresponding attention weight and sums them up to obtain the final output vector, as shown in Eq. 11:

$$A\left(x_{i}\right)=\sum_{j=1}^{n}w_{ij}V_{j}=\sum_{j=1}^{n}softmax\left(\frac{Q_{i}∙{K_{j}}^{T}}{\sqrt{d_{K}}}\right)V_{j}$$ (11)

where $A\left(x_{i}\right)$ represents the final output vector of the i -th element. For the sequence $X=\left[x_{1},x_{2},\cdots ,x_{n}\right]$ , its output vector is calculated as shown in Eq. 12:

$$A\left(X\right)=softmax\left(\frac{{QK}^{T}}{\sqrt{d_{K}}}\right)V$$ (12)

where X represents the input sequence, Q,K,V represent the results of linear transformations mapping X to the query, key, and value spaces respectively, $d_{K}$ is the dimension of the key vectors, and softmax is the normalization function.

In MHSA, multiple heads will produce multiple final output vectors. These output vectors can be concatenated or averaged to produce the final MHSA representation. This representation can be used for various downstream tasks such as semantic understanding, named entity recognition, machine translation (Zhou et al., 2023), etc. The final MHSA representation is shown as Eq. 13:

$$MHSA\left(X\right)=Concat\left(A_{1}\left(X\right),A_{2}\left(X\right),\cdots \cdots ,A_{n}\left(X\right)\right)$$ (13)

**FIGURE 5:**
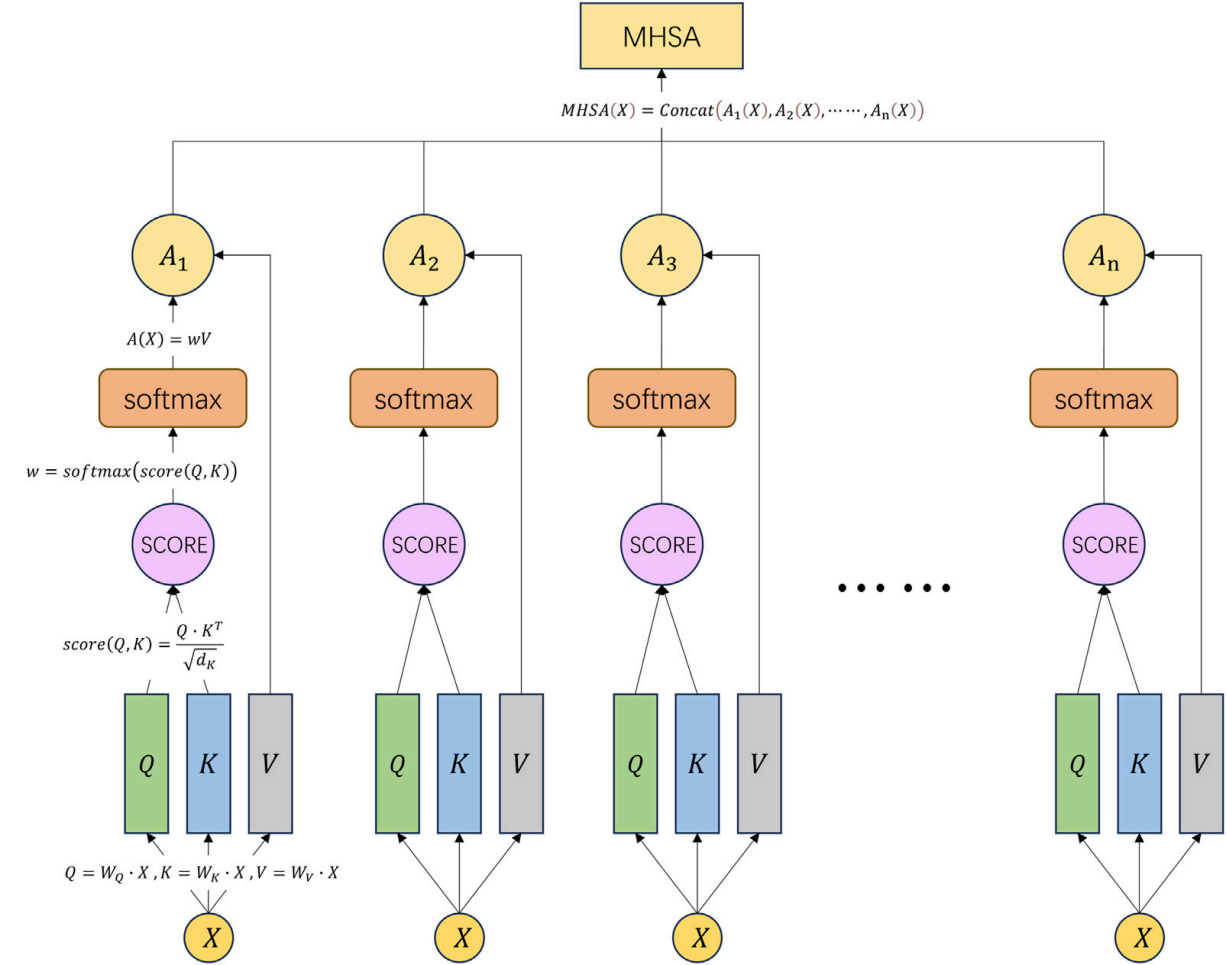
Schematic diagram of the multi-head self-attention mechanism.

We can refer to Figure 5 to understand the above formulas and computation process.

### 2.10 Classifier and training

During both the training and inference stages of this model, we concatenated the final feature vectors of the drugs and targets. Then, we passed the concatenated vector through a fully connected layer and applied the sigmoid function to obtain the final predicted value of the result. The calculation formula for the fully connected layer is shown as Eq. 14:

$$Z=W_{output}\left[y_{drug}\left\|y_{protein}\right.\right]+b_{output}$$ (14)

where $W_{output}$ represents the weight matrix, $b_{output}$ represents the bias vector, and $\left[∙\left\|∙\right.\right]$ denotes the concatenation operation applied to the two feature vectors. The sigmoid function is shown as Eq. 15:

$$Sigmoid\left(x\right)=\frac{1}{1+e^{-x}}$$ (15)

The mean squared error (MSE) function was selected as the loss function for this model due to the desired final computed result being a continuous value within the range of 0–1. The MSE stands as a widely adopted loss function utilized to quantify the disparity between predicted values and true values. It assesses the discrepancy by calculating the sum of squared differences between the predicted values and the true values, subsequently dividing it by the number of samples to acquire the squared average error. A smaller MSE signifies a reduced disparity between the predicted values and the true values, indicating a more favorable fit of the model. The calculation formula is represented as Eq. 16:

$$MSE=\frac{1}{N}\sum_{i=1}^{N}{\left(x_{i}-\;y_{i}\right)}^{2}$$ (16)

Where N represents the total number of training samples, $x_{i}$ and $y_{i}$ are the predicted and true values of the i -th training sample, respectively.

### 2.11 MIFAM-DTI model

In this study, a drug-target interaction prediction model named MIFAM-DTI was proposed, which leveraged multi-source information fusion and attention mechanisms. The model consisted of four main sections.

The first section involved the extraction of features for both drugs and targets. For drugs, the PCP feature vectors (refer to Supplementary Table S3) and the MACCS molecular fingerprint feature vectors (refer to Supplementary Table S4) were computed based on their SMILES sequences, utilizing the RDKit tool. For targets, the DC feature vectors (refer to Supplementary Table S5) and the ESM-1b feature vectors (refer to Supplementary Tables S6, S7) were calculated based on their protein amino acid sequences.

The second section of the study encompassed the fusion of feature vectors and adjacency matrices. Regarding the two types of feature vectors for drugs, an initial step involved utilizing PCA to reduce their dimensions to 128. Subsequently, cosine similarity matrices were computed for each type of feature vector. Employing PCA for dimensionality reduction served to denoise the data, eliminate redundant information, and enhance the accuracy of subsequent analyses. Furthermore, it aided in reducing the complexity associated with data storage and computation (Hussain et al., 2019). Following this, the reduced-dimensional PCP feature vectors and MACCS feature vectors were concatenated, resulting in fused drug feature vectors. The PCP cosine similarity matrix and MACCS cosine similarity matrix were then combined using a logical OR operation, yielding the fused drug adjacency matrix. The same operations were repeated for the two types of target feature vectors to obtain the fused target feature vectors and the fused target adjacency matrix.

The third section of the study centered around the utilization of the graph attention network and multi-head self-attention framework. At this stage, the fused drug feature vectors and adjacency matrices obtained from the second section were fed into the GAT. Within the multiple layers of the GAT, concatenation was performed on the various computed features, resulting in a feature vector denoted as $V_{GAT1}$ with dimensions $N_{layer}\times N_{\dim-GAT}$ . This approach allowed us to extract and retain more important information from the input vectors. Next, we fed the feature vector $V_{GAT1}$ into the MHSA module. For the multiple heads of MHSA, we calculated multiple features and then averaged them. We took the average of these new feature vectors together with the feature vector $V_{GAT1}$ to obtain the feature vector $V_{MHSA1}$ . Thus, we considered the combination of GAT and MHSA as a single entity. Next, we re-input $V_{MHSA1}$ and the drug adjacency matrix into another combination of GAT and MHSA. After performing the computations, we obtained the final feature vector for drugs. Similarly, for the fused feature vectors and adjacency matrix of targets, we repeated the above operations. After going through two layers of the GAT and MHSA combination, we finally obtained the final feature vector for targets.

The fourth section encompassed the final prediction stage of the model. In this stage, a multilayer perceptron was constructed, consisting of three fully connected layers. The output of the fully connected layers was then passed through the sigmoid function to obtain probability values between 0 and 1. During this stage, the final feature vectors obtained from the third section were concatenated for both drugs and targets. Subsequently, these concatenated feature vectors were fed into the MLP to predict the final interaction scores.

**FIGURE 6:**
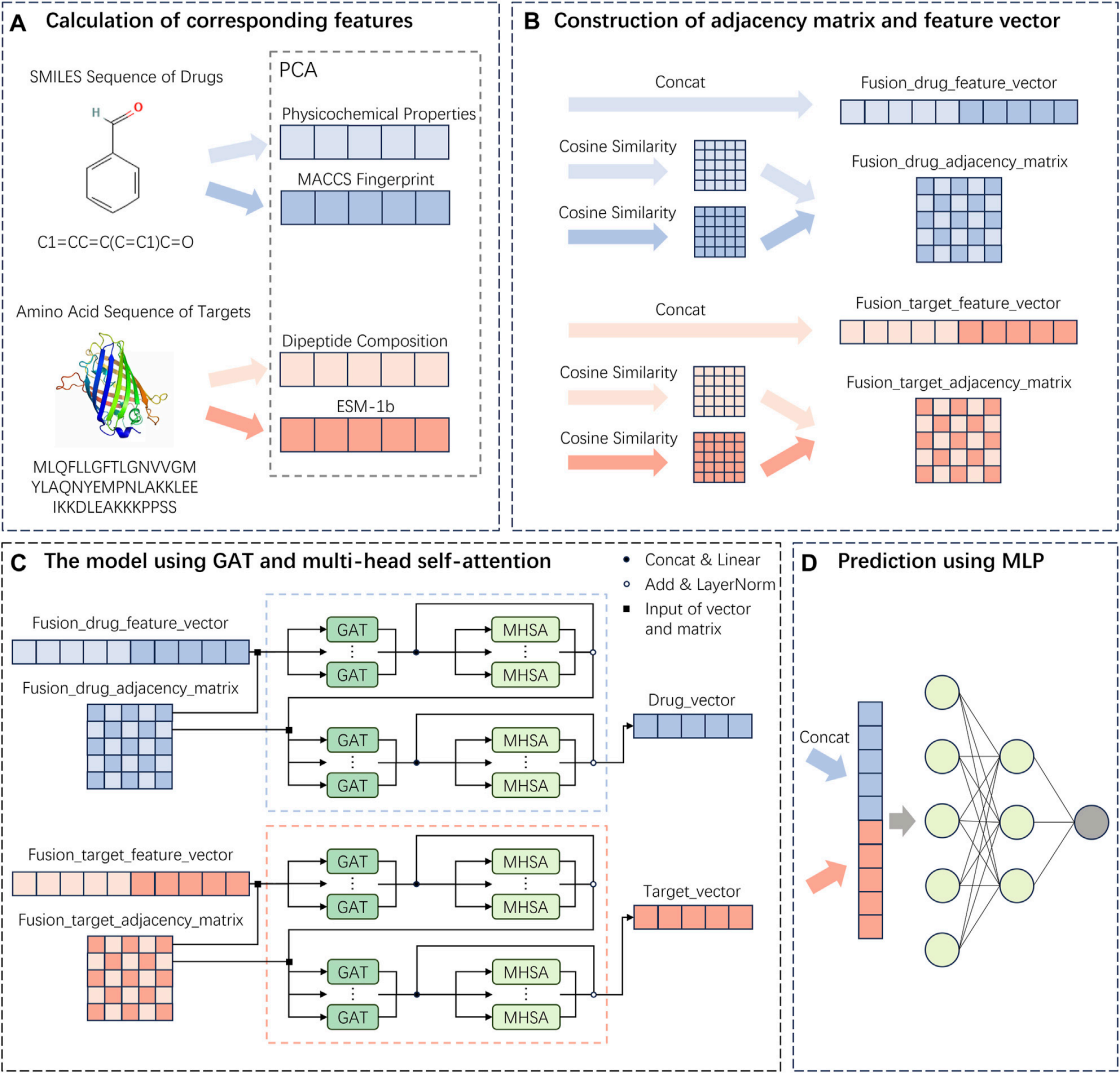
Flowchart of the MIFAM-DTI model. **(A)** Extraction of drug and target feature vectors. **(B)** The concatenation of feature vectors and the calculation of adjacency matrices. **(C)** Construction of deep learning model using GAT and MHSA. **(D)** Prediction of interaction scores using a MLP composed of fully connected layers.

The descriptions for each section of the MIFAM-DTI model flowchart shown in Figure 6 are as follows: (A) Drug and target feature extraction stage. (B) Feature vector fusion and adjacency matrix fusion stage. (C) GAT and MHSA framework. (D) Interaction score prediction stage. For more detailed information, please refer to Figure 6.

### 2.12 Configuration and parameters

The MIFAM-DTI model was based on the PyTorch (GPU) framework and utilizes Python 3.7.13 and CUDA 11.4. The implementation of this study was carried out on the CentOS 6.5 operating system, with an Intel(R) Xeon(R) CPU E5-2660 v3 @ 2.60 GHz processor. The total memory capacity is 189GB, and the GPU used is the NVIDIA Tesla V100S-PCI 32GB.

In this study, an ArgumentParser object was instantiated to define command line arguments and options. The model was optimized using the MSE loss function and the Adam optimizer. MSE loss function is widely employed for regression tasks as it facilitates the comparison of continuous numerical values between model outputs and target values. The Adam optimizer, a popular gradient descent optimization algorithm, adjusts the values of model parameters based on gradient information from the loss function (Yaqub et al., 2020). Its objective is to enhance the model’s fit to the training data and overall performance. The hyperparameter settings for this model can be found in Table 2.

**TABLE 2:** Default parameter settings of MIFAM-DTI.

Parameters	Value
Epochs	15
Patience	5
The learning rate	1e-4
Number of batch size	128
Number of hidden units	16
Number of layers of GAT	4
Number of heads of MHSA	4
Alpha for the leaky_relu	0.2
Activation Function (FC)	ReLU
Activation Function (Output)	Sigmoid
Loss function	MSELoss
Optimizer	Adam

## 3 Results

In this study, model training for the MIFAM-DTI model was conducted on two datasets, namely, *C. elegans* and Human. The initial step involved the extraction of feature vectors separately for drugs and targets. Subsequently, the data was utilized as input for the MIFAM-DTI model, enabling the generation of final drug-target interaction prediction scores. To assess the effectiveness of the model, a comparison was made with results obtained from current state-of-the-art methods, resulting in the demonstration of superior performance. The rationality and significance of each module within the model were validated through ablation experiments. Additionally, the practical applicability of the MIFAM-DTI model was demonstrated through case studies.

### 3.1 Results on the *C. elegans* and human datasets

To evaluate the performance of MIFAM-DTI, we conducted 10-fold cross-validation on the *C. elegans* and Human datasets, using the values of AUC, AUPR, Precision, Recall and F1-score as the main indicators of performance, and averaged the experimental results as the final results of the experiment. Table 3 shows the experimental results of MIFAM-DTI and compares them with seven mainstream methods, DrugE-Rank, RWR, DeepCPI, DeepConv-DTI, MHSADTI, TransformerCPI and AMMVF-DTI. The font of Table 3 represents the highest value.

**TABLE 3:** The results on all the dataset.

Datasets	Methods	AUC	AUPR	Precision	Recall	F1-score
C.elegans	DrugE-Rank	0.8221	0.8322	0.7906	0.7474	0.7684
	RWR	0.8493	0.8212	0.7860	0.7128	0.7475
	DeepCPI	0.9758	0.9571	0.9393	0.9271	0.9394
	DeepConv-DTI	0.9782	0.9711	0.9435	0.9423	0.9579
	MHSADTI	0.9838	0.9832	0.9465	0.9451	**0.9763**
	TransformerCPI	0.9880	—	0.9520	0.9530	—
	AMMVF-DTI	0.9900	—	0.9620	0.9600	—
	MIFAM-DTI	**0.9964**	**0.9956**	**0.9862**	**0.9625**	0.9742
Human	DrugE-Rank	0.8562	0.8257	0.7181	0.8668	0.7851
	RWR	0.8375	0.8165	0.7707	0.7243	0.7466
	DeepCPI	0.9692	0.9399	0.9187	0.9210	0.9096
	DeepConv-DTI	0.9738	0.9437	0.9295	0.9175	0.9204
	MHSADTI	0.9822	0.9568	0.9472	0.9365	0.9346
	TransformerCPI	0.9730	—	0.9160	0.9250	—
	AMMVF-DTI	0.9860	—	0.9760	**0.9380**	—
	MIFAM-DTI	**0.9874**	**0.9876**	**0.9762**	0.9145	**0.9443**

On the *C. elegans* dataset, the values of AUC, AUPR, Precision, Recall and F1-score achieved by the MIFAM-DTI model are 0.9964, 0.9956, 0.9862, 0.9625 and 0.9742, respectively. These results indicated that the model effectively discriminates between positive and negative samples in the dataset, exhibiting high prediction accuracy. Moreover, the values of AUC, AUPR, Precision and Recall of the MIFAM-DTI model surpassed those of the other mainstream methods. Only the F1-score indicator was slightly below the MHSADTI model of 0.0021. Specifically, the AUC value was higher than DrugE-Rank, RWR, DeepCPI, DeepConv-DTI, MHSADTI, TransformerCPI, and AMMVF-DTI by 0.1743, 0.1471, 0.0206, 0.0182, 0.0126, 0.0084, and 0.0064, respectively. Similarly, the AUPR value was higher by 0.1634, 0.1744, 0.0385, 0.0245, and 0.0124, respectively.

On the Human dataset, the MIFAM-DTI model achieved values of 0.9874 for AUC, 0.9876 for AUPR, 0.9762 for Precision, and 0.9443 for F1-score, outperforming the other seven mainstream methods. However, the value of Recall was 0.9145, which was lower than some mainstream models. Importantly, the AUPR value significantly improved from 0.9568 to 0.9876. Overall, the AUC value of the MIFAM-DTI model exceeded DrugE-Rank, RWR, DeepCPI, DeepConv-DTI, MHSADTI, TransformerCPI, and AMMVF-DTI by 0.1312, 0.1499, 0.0182, 0.0136, 0.0052, 0.0144, and 0.0014, respectively. Additionally, the AUPR value was higher by 0.1619, 0.1711, 0.0477, 0.0439, and 0.0308, respectively.

Combined with the aforementioned analysis, the MIFAM-DTI model demonstrated effective prediction of drug-target interactions. We attributed its exceptional performance to three key factors. First, the model incorporated the physicochemical properties of drugs along with the MACCS molecular fingerprint properties, DC properties of targets, and ESM-1b properties. This fusion approach better preserved the original properties of drugs and targets, enhancing the model’s predictive capability. Second, the MIFAM-DTI model utilized graph attention network and multi-head self-attention. GAT is adept at capturing local and global relationships in graph data by flexibly learning the importance among nodes through the attention mechanism (Gu et al., 2021). Since drug-protein interactions are often influenced by neighboring nodes, GAT is particularly suitable for tasks such as drug-protein interaction prediction. Alternatively, MHSA is employed to process sequence and graph data, leveraging the self-attention mechanism to learn dependencies among features. In drug-protein interaction prediction, where drug and protein features often contain rich sequence information, MHSA helps the model capture long-term dependencies in these sequences, thereby improving prediction accuracy (Jin et al., 2024). The simultaneous use of GAT and MHSA enabled comprehensive capture of complex relationships, leveraging their respective strengths on different data structures. This combination enhanced the model’s expressiveness and predictive performance, making it valuable in drug discovery, protein interactions, and related tasks. Third, the MIFAM-DTI model incorporated a two-layer GAT and MHSA ensemble. This architecture facilitated multi-level feature learning, enabling the gradual acquisition of higher-level feature representations and improving prediction accuracy (Kang et al., 2021). Furthermore, the sequential utilization of GAT and MHSA allowed for the integration of their modeling capabilities and characteristics, leveraging the advantages of each to enhance the model’s robustness and generalization ability.

Overall, the MIFAM-DTI model’s effectiveness stemmed from its fusion of multi-source information, the utilization of GAT and MHSA, and the incorporation of a two-layer GAT and MHSA ensemble. These factors collectively contributed to its enhanced expressiveness, improved predictive performance, and suitability for various applications in drug discovery, protein interactions, and related tasks.

### 3.2 Ablation experiments on PCA dimensionality reduction

By judiciously utilizing PCA (Principal Component Analysis) technology, it is possible not only to transform high-dimensional data into low-dimensional data, removing redundant information while preserving the main information of the data, but also to reduce the memory space required for storing data and lower the computational complexity of the model. To verify the impact of applying PCA on the model’s prediction results, we conducted a series of ablation experiments on the dimensions reduced by PCA. Since the dimension of the MACCS molecular fingerprint feature vector of drugs was 167, we set the dimensions of PCA dimensionality reduction to 128, 64, 32 and non-dimensionality reduction groups, respectively. In addition, the dimensionality of PCA reduction was set to integer powers of 2, and these numbers were more efficient to process in computer hardware, helping to optimize the performance and practicality of the model while adapting to the requirements of modern computing architectures. The experimental results are shown in Table 4. The highest values were indicated in bold black.

**TABLE 4:** The results of the different dimensions reduced by PCA.

Datasets	Dimensions	AUC	AUPR	Precision	Recall	F1-score
*C. elegans*	None	0.9957	0.9939	0.9660	0.9552	0.9606
	128	**0.9964**	**0.9956**	**0.9862**	0.9625	**0.9742**
	64	0.9959	0.9941	0.9485	**0.9699**	0.9591
	32	0.9920	0.9886	0.9416	0.9416	0.9416
Human	None	0.9776	0.9775	0.9167	**0.9395**	0.9279
	128	**0.9874**	**0.9876**	**0.9762**	0.9145	**0.9443**
	64	0.9808	0.9786	0.9385	0.9208	0.9295
	32	0.9801	0.9739	0.9267	0.9182	0.9224

In Table 4, “None” indicates that PCA was not used for dimensionality reduction, while 128, 64, and 32 represent the dimensions to which the input features were reduced using PCA. According to the experimental results, the model achieved favorable experimental outcomes on two datasets when the input features were reduced to 128 dimensions using PCA. In the *C. elegans* and Human datasets, when the dimensionality reduction of PCA was set to 128, the experimental results for AUC, AUPR, Precision, and F1-score all reached the maximum values among the conditions tested, with only Recall being slightly lower than in other dimensional settings. The comparison between “None” and 128 dimensions indicated that reducing the dimensionality of input features through PCA removed noise data and preserved the important parts, thereby enhancing the model’s predictive performance. However, when the dimensionality reduced by PCA was changed from 128 to 32, the model’s predictive performance decreased. This could be due to the loss of feature information in the original input caused by PCA dimensionality reduction, which in turn led to a decline in the model’s predictive capability.

### 3.3 Ablation experiments on multi-source information fusion

**FIGURE 7:**
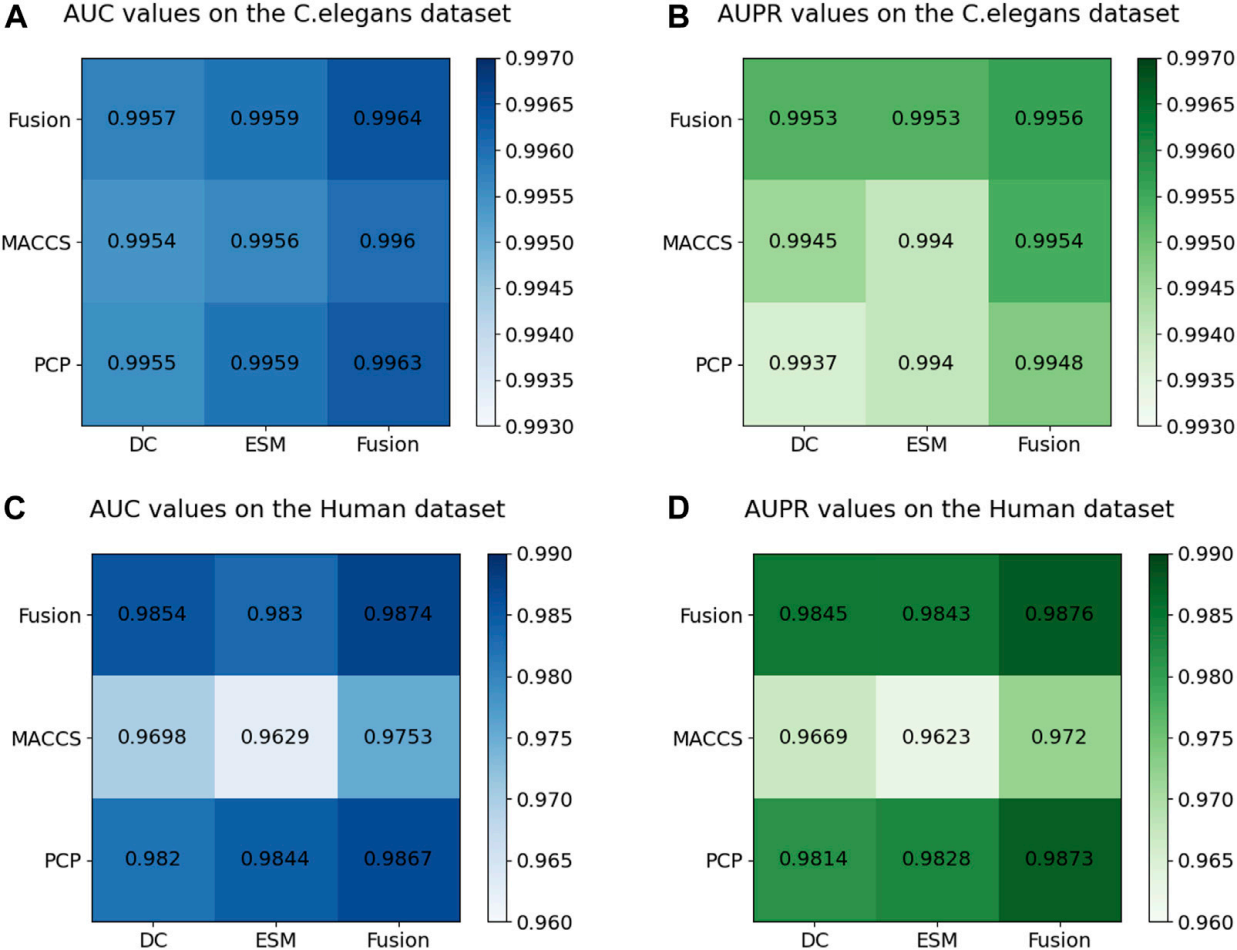
Results of ablation experiments on multi-source information fusion. **(A)** The AUC values on the *C. elegans* dataset. **(B)** The AUPR values on the *C. elegans* dataset. **(C)** The AUC values on the Human dataset. **(D)** The AUPR values on the Human dataset.

Figure 7 displays the AUC and AUPR values of the MIFAM-DTI model across different data characteristics. Subplots (A) and (B) represent the AUC and AUPR values on the *C. elegans* dataset, respectively. It is evident that the experimental results obtained through the fusion of drug characteristics and target characteristics were optimal. This outcome served as conclusive evidence of the model’s effectiveness in leveraging the fusion of multi-source information. Subplots (C) and (D) illustrate the AUC and AUPR values of the model on the Human dataset. Despite the MACCS molecular fingerprint feature vector of the drug exhibiting less impressive performance on this dataset, the experimental results following the fusion of both drug features still surpassed those of the single feature. This outcome further validated the rationale behind the fusion of multi-source information. Moreover, the robust and stable performance of the multi-source information fusion-based approach on both datasets demonstrated the model’s resilience and stability.

### 3.4 Ablation experiments on the number of layers of GAT and MHSA complexes

Table 5 provides the outcomes of employing different numbers of layers in the GAT and MHSA combination. The highest values were indicated in bold black. The models were denoted as MIFAM-DTI- i , where i corresponded to the number of layers in the GAT and MHSA combination. The results showed that for both the *C. elegans* and Human datasets, the AUC, AUPR, Precision, Recall and F1-score values were maximized when i equaled 2. This finding suggested that the best experimental performance was achieved when utilizing a GAT and MHSA combination with two layers. For instance, considering the *C. elegans* dataset, as the number of layers in the combination increased from 1 to 2, the AUC, AUPR, Precision, Recall and F1-score values rose by 0.0004, 0.0010, 0.0411, 0.0062 and 0.0235, respectively. This observation indicated that through multi-layer feature learning, deep learning models could extract more representative feature representations from the raw input data, resulting in improved model performance and generalization (Parvandeh et al., 2019). However, when the number of layers increased from 2 to 3, both the AUC, AUPR, Precision, Recall and F1-score values declined. This decline may be attributed to the excessive utilization of GAT and MHSA, wherein their attention mechanisms might focus more on noise and irrelevant features within the training data, consequently leading to overfitting of the model (Kim and Simon, 2014).

**TABLE 5:** The results of the GAT and MHSA combination with different layers.

Datasets	Layers	AUC	AUPR	Precision	Recall	F1-score	Time (s)
C.elegans	MIFAM-DTI-1	0.9960	0.9946	0.9451	0.9563	0.9507	40.71
	MIFAM-DTI-2	**0.9964**	**0.9956**	**0.9862**	**0.9625**	**0.9742**	76.10
	MIFAM-DTI-3	0.9925	0.9884	0.9618	0.9438	0.9527	108.52
Human	MIFAM-DTI-1	0.9678	0.9622	0.9237	0.8996	0.9115	57.36
	MIFAM-DTI-2	**0.9874**	**0.9876**	**0.9762**	**0.9145**	**0.9443**	111.00
	MIFAM-DTI-3	0.9767	0.9753	0.9357	0.9065	0.9209	162.53

### 3.5 Case studies of coenzymes involved in cellular energy metabolism

To further demonstrate and analyze the predictive ability of the MIFAM-DTI model, we selected two coenzyme-like substances related to cellular energy metabolism, NADH (Li et al., 2009) and Adenosine-5′-triphosphate (Szewczyk and Pikuła, 1998), as validation targets from the Human dataset. We screened twenty targets (Q99714, P07195, P03897, P51649, Q9UI09, P16083, P20839, Q04828, P09622, P51970, P09601, O43920, P40926, Q06278, P14679, Q16878, P49448, Q08426, P31937, and O60701) that interact with NADH from the PubChem database and UniProt database. Additionally, we selected sixteen targets (P42684, Q07912, Q04771, P00414, P00734, Q02880, Q12888, Q5S007, Q9Y2U5, P08253, P14780, P35228, Q12809, P37231, P53041, and P35354) that interact with Adenosine-5′-triphosphate. None of these drug targets pairs are annotated as interacting in the Human dataset. Additionally, we selected 4-Epitetracycline hydrochloride and 5-HT3 antagonist 3 as control groups for NADH and Adenosine-5′-triphosphate, respectively. Database review confirmed that there were no known interactions between 4-Epitetracycline hydrochloride and the twenty corresponding targets, nor between 5-HT3 antagonist 3 and the sixteen corresponding targets.

**FIGURE 8:**
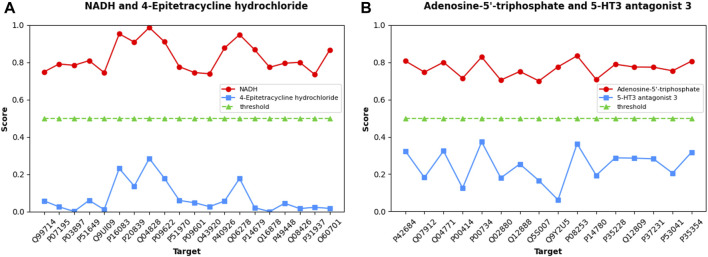
Predicted scores of DTIs about Coenzyme substances using our MIFAM-DTI model. In figure **(A)**, the red circle represents the interaction between NADH and its 20 targets, and the blue square represents the interaction between the unrelated drug 4-Epitetracycline hydrochloride and the same targets. In figure **(B)**, the red circle represents the interaction between Adenosine-5′-triphosphate and its 16 targets, and the blue square represents the interaction between the unrelated drug 5-HT3 antagonist 3 and the same targets. In both graphs, the green triangle is the threshold, set to 0.5.

Initially, the MIFAM-DTI model was trained on the Human dataset while preserving all its parameters. Subsequently, the SMILES sequences of four drugs were obtained from the PubChem website, and the amino acid sequences of the aforementioned thirty-six targets were retrieved from the UniProt database. After calculating the corresponding feature vectors, the data was inputted into the pre-trained MIFAM-DTI model for interaction prediction. The interaction prediction scores between the four drugs and their respective targets are presented in Figure 8.

Figure 8A exhibits the prediction scores of interactions between NADH and 4-Epitetracycline hydrochloride with twenty targets, while Figure 8B showcases the predicted scores of interactions between Adenosine-5′-triphosphate and 5-HT3 antagonist 3 with sixteen targets. The X-axis represents the UniProt ID of each target, and the Y-axis denotes the predicted scores of interactions between drug-target pairs. In Figures 8A, B, NADH and Adenosine-5′-triphosphate are depicted as red circles, while control group 4-Epitetracycline hydrochloride and 5-HT3 antagonist 3 are represented by blue squares. The green triangle signifies the initial threshold, set at 0.5 in this case. The results demonstrate that the predicted scores of the two coenzyme substances and their corresponding targets surpassed the threshold, while the predicted scores of the control drugs and their corresponding targets fall below the threshold. This observation indicated the model’s proficient forecasting ability. Although some values in the prediction scores of NADH and Adenosine-5′-triphosphate may not be notably high, they still exceed the threshold in this case without significant bias. Hence, the model’s prediction outcomes remained within an acceptable range.

Consequently, the following steps should be followed when employing the MIFAM-DTI model. Firstly, training on an extensive dataset of drug-target interactions is necessary to comprehend the general relationship within such interactions. The drug input requires the provision of the SMILES sequence, while the target input necessitates the amino acid sequence of the protein. Secondly, information regarding the drug and target should be acquired and screened from existing databases, followed by preprocessing of the data and inputting it into the MIFAM-DTI model for interaction score prediction. Lastly, the predicted scores of the drug and target serve as a reference standard for identifying candidate drugs that may impact the target or possess potential therapeutic effects on the target. It is important to note that the prediction score derived from the deep learning model MIFAM-DTI is merely an auxiliary tool in drug development and cannot be solely relied upon for judging drug-target interactions. To ensure the drug’s efficacy, it is imperative to conduct biological experiments and clinical trials on the candidate drug. Upon approval by regulatory authorities, the drug can be utilized for treating the target disease, ultimately concluding the drug development process.

## 4 Conclusion and discussion

Accurate prediction of drug-target interactions is crucial for enhancing drug development efficiency and mitigating unknown risks (Wang et al., 2022). Despite the increasing number of DTI prediction methods and their improved accuracies, there is still considerable room for further improvement.

In this study, a deep learning model named MIFAM-DTI was proposed, which leveraged multi-source information fusion and attention mechanisms to predict DTIs. MIFAM-DTI integrated multiple sources of characteristic information from drugs and targets, thereby preserving more original information. Through graph attention network and multi-head self-attention, MIFAM-DTI effectively extracted and retained important features while eliminating or reducing unnecessary ones, resulting in the final feature vectors for drugs and targets. These feature vectors were combined and fed into a multi-layer perceptron constructed by fully connected layers to obtain the final prediction probability. The performance of the proposed model was evaluated using 10-fold cross-validation, demonstrating improved prediction results.

Although MIFAM-DTI had shown promising experimental results, it still exhibited certain limitations that required further refinement. Firstly, while the fusion of multi-source information of drugs and targets had been considered, the ever-increasing availability of such information necessitated careful selection, as the current approach might be biased (Kennedy et al., 2022). Furthermore, the characteristic information employed in this method was derived solely from one-dimensional data, such as drug SMILES and amino acid sequences of targets. In the future, incorporating additional feature information into deep learning models is being contemplated (Lv et al., 2023). Secondly, the adjacency matrix information used in the model was obtained through cosine similarity calculations among feature vectors. Incorporating network information, such as drug-drug interaction networks and target-target interaction networks, is anticipated to enhance the accuracy of DTI prediction. Lastly, the method employed multiple GAT and MHSA, resulting in a substantial number of parameters. During the model training phase, this led to challenges such as extended training time and high memory consumption. To address these issues, future research can focus on optimizing the model structure and reducing its time and space complexity (Stahlschmidt et al., 2022).

## Data Availability

The original contributions presented in the study are included in the article/Supplementary Material, further inquiries can be directed to the corresponding author.

## Supplementary material

SUPPLEMENTARY TABLE S1
*C. elegans* Datasets.

SUPPLEMENTARY TABLE S2 Human Datasets.

SUPPLEMENTARY TABLE S3 The PCP feature vector of drugs.

SUPPLEMENTARY TABLE S4 The MACCS molecular fingerprint feature vector of drugs.

SUPPLEMENTARY TABLE S5 The DC feature vector of targets.

SUPPLEMENTARY TABLE S6 The ESM-1b feature vector of targets in *C. elegans*.

SUPPLEMENTARY TABLE S7 The ESM-1b feature vector of targets in Human.
